# Pazopanib versus sunitinib in Chinese patients with locally advanced or metastatic renal cell carcinoma: pooled subgroup analysis from the randomized, COMPARZ studies

**DOI:** 10.1186/s12885-020-6708-8

**Published:** 2020-03-14

**Authors:** Xinan Sheng, Jie Jin, Zhisong He, Yiran Huang, Aiping Zhou, Jinwan Wang, Xiubao Ren, Dingwei Ye, Xu Zhang, Shukui Qin, Fangjian Zhou, Binhui Wang, Jun Guo

**Affiliations:** 1 Key Laboratory of Carcinogenesis and Translational Research (Ministry of Education/Beijing), Department of Renal Cancer and Melanoma, Peking University Cancer Hospital & Institute, Beijing, China; 2 Peking University First Hospital, Beijing, China; 3 Ren Ji hospital affiliated to Shanghai Jiao Tong University, Shanghai, China; 4 Cancer Hospital, CAMS & PUMC, Beijing, China; 5 Tianjin Cancer Hospital, Tianjin, China; 6 Cancer Hospital Affiliated to Fudan University, Shanghai, China; 7 Chinese Beijing 301 PLA Hospital, Department of Urology, Beijing, China; 8 PLA Cancer Center, Nanjing Bayi Hospital, Nanjing, China; 9 Sun Yat-Sen University Cancer Center, Guangzhou, China; 10 Novartis Oncology, Beijing, China

**Keywords:** Renal cell carcinoma, Pazopanib, Sunitinib, Chinese

## Abstract

**Background:**

We performed a pooled analysis of the COMPARZ study assessing efficacy and safety of pazopanib versus sunitinib in treatment-naïve Chinese patients with locally advanced and/or metastatic renal cell carcinoma (a/mRCC).

**Methods:**

In the COMPARZ study, patients were randomized (1:1) to receive pazopanib 800 mg once daily (QD) continuously or sunitinib 50 mg QD in 6-week cycles (4 weeks on, 2 weeks off). The primary endpoint was progression-free survival (PFS); secondary endpoints included overall response rate (ORR), overall survival (OS), and safety. PFS and ORR were assessed by independent review committee (IRC) and local investigators.

**Results:**

Of the 209 Chinese patients (pazopanib, [*n* = 109] and sunitinib, [*n* = 100]), 155 (74%) were males and median age was 57 years (range, 18–79). Median PFS was 13.9 months for pazopanib versus 14.3 months for sunitinib per investigator assessment and 8.3 months in both arms per IRC assessment; PFS hazard ratio was 1.17 (investigator) and 0.99 (IRC). Median OS was not reached in pazopanib arm and was 29.5 months in sunitinib arm. ORR was significantly higher in pazopanib arm versus sunitinib arm (investigator: 41% versus 23% [*P* = 0.0052]; IRC: 35% versus 20% [*P* = 0.0203]). Pazopanib was generally well tolerated in Chinese patients with a/mRCC. Most frequent AEs in the pazopanib arm were diarrhea and hair color changes whereas the most frequent AEs in the sunitinib arm were decreased platelets, decreased neutrophil count, and thrombocytopenia.

**Conclusion:**

The results of the pooled analysis were consistent with the overall population in the COMPARZ study, and confirmed similar PFS and OS of pazopanib and sunitinib in the Chinese patients.

**Trial registration:**

clinical trials.gov, NCT00720941 (August 14, 2008) and NCT01147822 (May 19, 2010).

## Background

Renal cell carcinoma (RCC) is the most common form of kidney cancer (approximately 90%) with clear cell RCC constituting approximately 75 to 80% of RCC [1]. As per the National Cancer Registry of China, there were 45,096 new RCC cases in 2011, accounting for 1.34% of all malignancies. RCC accounted for 0.5% of all cancer deaths and ranked 16th among all cancers [2]. According to the Chinese Cancer Registry’s annual report of 2015, the incidence and mortality of RCC were higher in males versus females, (male/female ratio of 2:1) and also higher in urban areas than in rural areas [2].

In China, the approved agents for the treatment of metastatic RCC include the tyrosine kinase inhibitors (TKIs; pazopanib, sunitinib, sorafenib, axitinib) and the mammalian target of rapamycin (mTOR) inhibitor, everolimus [2]. In addition, there are ongoing studies for immune checkpoint inhibitors like nivolumab [3]. Pazopanib and sunitinib are first-line agents acting on the vascular endothelial growth factor receptors (VEGFRs) 1, 2, 3 as well as platelet-derived growth factor receptors and other tyrosine kinases [4]. Single agent TKI treatment remains important in China where pembrolizumab+axitinib and nivolumab+ipilimumab are not available. Differences have been reported in the efficacy and safety seen with TKIs in Chinese patients compared to Western patients [5–7].

The COMPARZ study evaluated the relative efficacy and safety profiles of first-line pazopanib and sunitinib in patients with advanced or metastatic RCC and demonstrated that that the efficacy of these drugs is comparable, but that there were significant differences in safety profiles and patient quality-of-life [8]. The objective of the pooled analysis from the COMPARZ study was to compare the efficacy and safety profiles of pazopanib and sunitinib in Chinese patients with locally advanced or metastatic RCC.

## Methods

Detailed eligibility criteria, study design, efficacy endpoints, and statistical methods of the COMPARZ trial have been reported previously [8].

### Patients

The key inclusion criteria were diagnosis of RCC with clear-cell component histology, locally advanced or metastatic disease, patients who received no prior systemic therapy (interleukin-2, interferon alpha, chemotherapy, bevacizumab, mTOR inhibitor, sunitinib, sorafenib, or other VEGF TKI) for advanced or metastatic RCC, measurable disease per Response Evaluation Criteria In Solid Tumors (RECIST) v1.0, Karnofsky performance scale value of ≥70, and adequate organ system functions.

The key exclusion criteria included history of another malignancy, history or clinical evidence of central nervous system metastases, poorly controlled hypertension, history of cardiovascular conditions, any serious and/or unstable preexisting medical, psychiatric, or other conditions that could interfere with patient’s safety, obtaining informed consent, or compliance to the study, prior use of an investigational or licensed drug that targets VEGF or VEGFRs (eg, bevacizumab, sunitinib, sorafenib, etc), or use of mTOR inhibitors (eg, temsirolimus, everolimus, etc).

### Study design

The COMPARZ study was a randomized, open-label, parallel-group, phase 3 trial, which evaluated the efficacy and safety of pazopanib versus sunitinib in patients with advanced or metastatic RCC. The study design has been reported previously [8]. NCT01147822 was designed as a substudy of NCT00720941 to compare the efficacy and safety of pazopanib versus sunitinib in Asian population [8]. In total, 209 Chinese patients were enrolled in the COMPARZ study. Eighty patients were enrolled in NCT00720941 from 10 Oct 2009 to 26 Apr 2010, and 129 patients were enrolled in NCT01147822 from 26 May 2010 to 30 Sep 2011. Written informed consent was obtained from each patient before performing any study-specific procedures.

Randomization was stratified for Karnofsky Performance Scale of 70–80 or 90–100, baseline levels of lactate dehydrogenase (> 1.5 versus ≤1.5 times upper limit of normal), and previous nephrectomy (yes versus no). Eligible patients were centrally randomized 1:1 to receive either pazopanib 800 mg once daily (QD) continuously or sunitinib 50 mg QD in 6-week cycles (4 weeks of treatment followed by 2 weeks without treatment). Patients received treatment until disease progression per investigator (RECIST 1.0), death, unacceptable toxicity, or consent withdrawal for any reason.

### Endpoints and assessments

The primary objective was to compare the progression-free survival (PFS) of patients treated with pazopanib versus sunitinib. The secondary objectives were to compare the overall survival (OS), overall response rate (ORR), time to response, duration of response (DOR), and safety in RCC patients treated with pazopanib versus sunitinib.

**Fig. 1:**
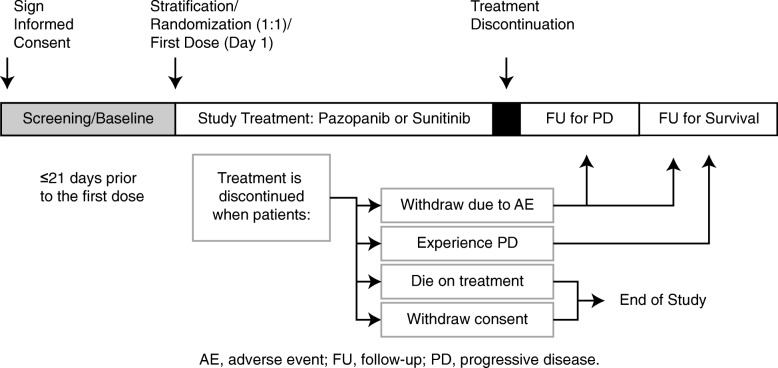
The COMPARZ study design. AE, adverse event; FU, follow-up; PD, progressive disease

Efficacy assessments were scheduled at screening/baseline with follow-up every 6 weeks till week 24, and then every 12 weeks thereafter until progressive disease (PD), death, unacceptable toxicity, or withdrawal of consent (Fig. 1). Computed tomography or magnetic resonance imaging data were evaluated by investigators and reevaluated by an independent review committee (IRC). Safety assessments were evaluated every 6 weeks until week 24, and every 12 weeks thereafter until progression of disease [8].

### Statistical analysis

The treatment HR for PFS analysis was estimated by a Cox model. For each treatment arm, the Kaplan-Meier survival curves were presented. A sensitivity analysis of IRC-assessed PFS was performed to explore the robustness of the results of the primary analysis. This sensitivity analysis was similar to the primary analysis except that the analysis did not use the stratification factors to adjust/stratify the analysis. Overall survival was summarized using Kaplan-Meier survival curves and compared between treatment arms using a log-rank test.

## Results

### Patients

A total of 230 patients were screened and 209 patients (109 randomized to pazopanib and 100 to sunitinib) were enrolled in China. The median age was 57 years (range, 18–79 years), with more men than women enrolled as expected for the disease population. The disease characteristics at initial diagnosis and at screening were balanced between the two treatment arms (Table 1), with the exception of median time since initial diagnosis, which was observed to be longer in the sunitinib arm (198 days; interquartile range [IQR]: 34, 984) than in the pazopanib arm (89 days; IQR: 30, 760). The most common disease locations at baseline were the lung, kidney, lymph nodes, and bone.

**Table 1:** Summary of baseline characteristics (Chinese ITT population)

	Pazopanib	Sunitinib	Total	***P*** value
**Number of patients**	109	100	209	
**Age, (years)**				0.8991
Mean (SD)	55.5 (11.57)	55.7 (11.16)	55.6 (11.35)	
Median (min, max)	58 (18, 76)	57 (23, 79)	57 (18, 79)	
**Sex, n (%)**				0.5611
Female	30 (28)	24 (24)	54 (26)	
Male	79 (72)	76 (76)	155 (74)	
**Weight (kg), n (%)**				0.7388
Mean (SD)	66.44 (12.665)	67.02 (12.414)	66.72 (12.519)	
Median (min, max)	67 (36, 110)	66 (40, 95)	67 (36, 110)	
**Primary tumor type, n (%)**				NA
Renal cell	109 (100)	100 (100)	209 (100)	
**Histology, n (%)**				0.4290
Clear cell	107 (98)	96 (96)	203 (97)	
Predominantly clear cell	2 (2)	4 (4)	6 (3)	
**Stage at screening, n (%)**				0.9151
I	1 (< 1)	0	1 (< 1)	
II	0	1 (1)	1 (< 1)	
III	3 (3)	3 (3)	6 (3)	
IV	104 (95)	96 (96)	200 (96)	
Missing	1 (< 1)	0	1 (< 1)	
**Metastatic disease at screening, n (%)**				0.3023
Yes	106 (97)	97 (97)	203 (97)	
No	2 (2)	3 (3)	5 (2)	
Missing	1 (< 1)	0	1 (< 1)	
**MSKCC risk category, n (%)**				0.1588
Favorable risk	26 (24)	34 (34)	60 (29)	
Intermediate risk	75 (69)	64 (64)	139 (67)	
Poor risk	6 (6)	2 (2)	8 (4)	
Unknown	2 (2)	0	2 (<1)	
**Heng risk category, n (%)**				0.2168
Favorable risk	24 (22)	33 (33)	57 (27)	
Intermediate risk	70 (64)	55 (55)	125 (60)	
Poor risk	14 (13)	12 (12)	26 (12)	
Unknown	1 (< 1)	0	1 (< 1)	

*ITT* Intent to treat, *MSKCC* Memorial Sloan-Kettering Cancer Center, *SD* Standard deviation

### Efficacy results

**Fig. 2:**
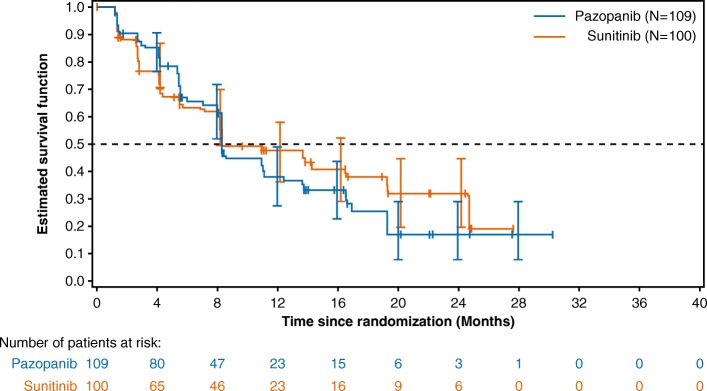
Kaplan-Meier progression-free survival curves

In the Chinese subgroup, the efficacy results of pazopanib were similar to sunitinib in terms of PFS, OS, and ORR. The IRC-assessed PFS HR was 0.9927 (95% CI, 0.6760–1.4580). The HR for the Chinese population was consistent with the IRC-assessed PFS for the overall population in the COMPARZ study (HR, 1.047; 95% CI, 0.8982–1.2195). The median PFS in the pazopanib arm (8.3 months; 95% CI, 8.2–11.1) was similar as that in the sunitinib arm (8.3 months; 95% CI, 8.1–19.3) (Fig. 2), which shows that the efficacy of pazopanib was similar to that of sunitinib in the Chinese population (Table 2).

**Table 2:** Efficacy assessments

Parameter	Investigator assessment		IRC assessment	
	Pazopanib	Sunitinib	Pazopanib	Sunitinib
**Number of patients**	109	100	109	100
**Median PFS (1st quartile, 3rd quartile) months**	13.9 (8.0, 20.2)	14.3 (5.6, 27.7)	8.3 (5.5, 19.3)	8.3 (4.1, 24.7)
**HR (95% CI)**	1.17 (0.792–1.727) *P* = 0.4381		0.99 (0.6760–1.4580) *P* = 0.9629	
**ORR, %**	41	23	35	20
	P = 0.0052		P = 0.0203	

*CI* Confidence interval, *HR* Hazard ratio, *IRC* Independent review committee, *ORR* Overall response rate, *PFS* Progression-free survival

The investigator-assessed PFS HR was 1.169 (95% CI, 0.792–1.727). The median PFS was 13.9 months for pazopanib versus 14.3 months for sunitinib. The results of the sensitivity analysis for PFS (HR 1.077, 95% CI: 0.740–1.569) were consistent with the results of the primary analysis suggesting that PFS was similar for pazopanib and sunitinib.

**Fig. 3:**
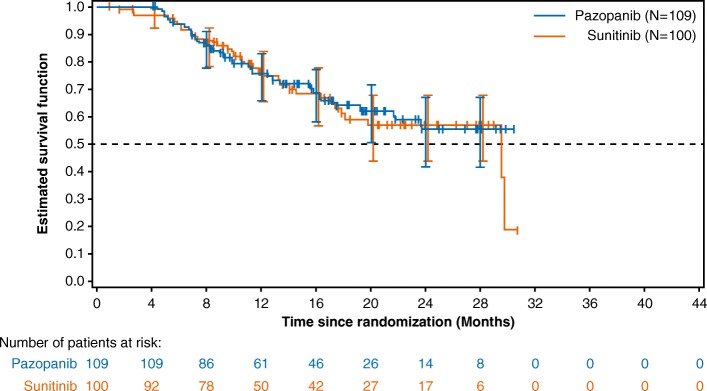
Kaplan-Meier overall survival curves

The OS was similar between the two treatment arms (Fig. 3). The HR for median OS was 0.938 (95% CI, 0.583–1.510; *P* = 0.792). The median OS was 29.5 months (IQR: 12.1, 29.8) in the sunitinib arm but was not yet reached in the pazopanib arm (IQR: 12.6, not reached).

The response rate (complete response [CR] + partial response [PR]) in the pazopanib arm was higher compared to the sunitinib arm based on IRC assessment (35% versus 20%, respectively) and the difference (15%) was statistically significant *(P =* 0.02). Consistent with the IRC-assessed response, the investigator-assessed response rate was also higher in the pazopanib arm than the sunitinib arm, and the difference (18%) was statistically significant *(P =* 0.005). The median time to IRC-assessed response was 11.9 weeks (IQR: 6.3, 18.0) in the pazopanib arm and 12.1 weeks (IQR: 11.3, 18.0) in the sunitinib arm. DOR data is inconclusive due to small number of responders in each arm (38/109 in pazopanib and 20/100 in sunitinib) and it is not statistically valid to compare them.

### Safety results

The most common adverse events (AEs; > 35% in either of the treatment arms) were hypertension, diarrhea, hand-foot syndrome (HFS), hair color changes, increased alanine aminotransferase (ALT), increased aspartate aminotransferase (AST), fatigue, decreased appetite, proteinuria, leukopenia, neutropenia, decreased neutrophil count, decreased platelet count, and thrombocytopenia; these AEs were consistent with those commonly observed for the class of VEGF TKI.

Of the AEs occurring in ≥10% of patients in either of the treatment arms (Table 3), the following AEs occurred more frequently (95% CI for relative risk excluding one, unadjusted for multiplicity) in the sunitinib arm compared to the pazopanib arm: increased blood creatinine, decreased white blood cell count, decreased neutrophil count, decreased platelets and thrombocytopenia, eyelid edema, decreased hemoglobin, increased blood lactate dehydrogenase, increased blood thyroid stimulating hormone, peripheral edema, stomatitis, anemia, nasopharyngitis, facial edema, yellow skin, and xanthochromia. AEs occurring more frequently (95% CI for relative risk excluding one, unadjusted for multiplicity) in the pazopanib arm compared to the sunitinib arm were diarrhea, hair color changes, and skin hypopigmentation (Table 4).

**Table 3:** Summary of on-therapy adverse events occurring in ≥10% of patients in either of the treatment arms (Chinese safety population)

Adverse event	Pazopanib ***N*** = 109 n (%)	Sunitinib ***N*** = 100 n (%)
Patients with any event	108 (> 99)	99 (99)
Hypertension	65 (60)	50 (50)
Diarrhea	57 (52)	37 (37)
Palmar-plantar erythrodysesthesia syndrome (HFS)	52 (48)	57 (57)
Hair color changes	47 (43)	13 (13)
Alanine aminotransferase increased	45 (41)	32 (32)
Fatigue	43 (39)	41 (41)
Aspartate aminotransferase increased	41 (38)	32 (32)
Decreased appetite	41 (38)	32 (32)
Proteinuria	39 (36)	39 (39)
Leukopenia	33 (30)	43 (43)
Blood bilirubin increased	27 (25)	21 (21)
Neutropenia	26 (24)	36 (36)
Neutrophil count decreased	25 (23)	40 (40)
Platelet count decreased	23 (21)	39 (39)
Blood creatinine increased	21 (19)	32 (32)
Thrombocytopenia	20 (18)	39 (39)
Nausea	19 (17)	15 (15)
Vomiting	19 (17)	9 (9)
Hypothyroidism	18 (17)	23 (23)
Mouth ulceration	17 (16)	25 (25)
White blood cell count decreased	17 (16)	33 (33)
Eyelid edema	16 (15)	28 (28)
Abdominal pain upper	15 (14)	7 (7)
Bilirubin conjugated increased	15 (14)	6 (6)
Blood bilirubin unconjugated increased	15 (14)	8 (8)
Hemoglobin decreased	13 (12)	31 (31)
Skin hypopigmentation	13 (12)	3 (3)
Blood triglycerides increased	12 (11)	14 (14)
Blood lactate dehydrogenase increased	9 (8)	18 (18)
Epistaxis	9 (8)	11 (11)
Hypogeusia	9 (8)	12 (12)
Pain in extremity	9 (8)	12 (12)
Rash	9 (8)	14 (14)
Blood thyroid stimulating hormone increased	8 (7)	19 (19)
Anemia	7 (6)	25 (25)
Dysgeusia	6 (6)	12 (12)
Peripheral edema	6 (6)	14 (14)
Blood cholesterol increased	5 (5)	10 (10)
Stomatitis	4 (4)	12 (12)
Facial edema	3 (3)	17 (17)
Yellow skin	3 (3)	22 (22)
Nasopharyngitis	2 (2)	10 (10)
Xanthochromia	1 (< 1)	10 (10)

*HFS* Hand-foot syndrome

**Table 4:** Summary of relative risk (95% CI excluding one) of adverse events occurring in ≥10% of patients in either of the treatment arms (Chinese safety population)

			Relative risk (pazopanib/sunitinib)		
Preferred term	Pazopanib (***N*** = 109)	Sunitinib (***N*** = 100)	Ratio	95% CI	***P*** value
Skin hypopigmentation	13 (12)	3 (3)	3.98	(1.167–13.543)	0.0270
Hair color changes	47 (43)	13 (13)	3.32	(1.912–5.755)	< 0.001
Diarrhea	57 (52)	37 (37)	1.41	(1.034–1.932)	0.0309
Blood creatinine increased	21 (19)	32 (32)	0.60	(0.373–0.972)	0.0362
Neutrophil count decreased	25 (23)	40 (40)	0.57	(0.377–0.872)	0.0086
Platelet count decreased	23 (21)	39 (39)	0.54	(0.349–0.838)	0.0059
Eyelid edema	16 (15)	28 (28)	0.52	(0.302–0.910)	0.0200
Thrombocytopenia	20 (18)	39 (39)	0.47	(0.295–0.750)	0.0016
White blood cell count decreased	17 (16)	33 (33)	0.47	(0.281–0.794)	0.0044
Blood lactate dehydrogenase increased	9 (8)	18 (18)	0.46	(0.216–0.974)	0.0429
Blood thyroid stimulating hormone increased	8 (7)	19 (19)	0.39	(0.177–0.843)	0.0179
Peripheral edema	6 (6)	14 (14)	0.39	(0.157–0.984)	0.0440
Hemoglobin decreased	13 (12)	31 (31)	0.38	(0.214–0.693)	0.0013
Stomatitis	4 (4)	12 (12)	0.31	(0.102–0.917)	0.0362
Anemia	7 (6)	25 (25)	0.26	(0.116–0.568)	< 0.001
Nasopharyngitis	2 (2)	10 (10)	0.18	(0.041–0.817)	0.0244
Facial edema	3 (3)	17 (17)	0.16	(0.049–0.536)	0.0052
Yellow skin	3 (3)	22 (22)	0.13	(0.039–0.405)	< 0.001
Xanthochromia	1 (< 1)	10 (10)	0.09	(0.012–0.704)	0.0203

*CI* Confidence interval

## Discussion

The study results suggest that both pazopanib and sunitinib can effectively improve the OS and PFS in Chinese patients with locally advanced or metastatic RCC in first-line treatment. The results of the Chinese subgroup analysis were mostly consistent with the overall population in the COMPARZ study.

Although the ORR in pazopanib arm was significantly higher than sunitinib arm, it did not translate into a PFS advantage over sunitinib. The IRC and investigator assessed median PFS values for the Chinese subgroup were similar in both arms. The difference between the IRC and the investigator-assessed PFS in terms of HR and median PFS could be due to the relatively small sample size in the Chinese subgroup and potential difference in judgment of tumor progression between the investigators and IRC. This is also not indicative that the investigators necessarily assess sunitinib as better given the small sample size and does not affect the consistency in terms of PFS as assessed by IRC between the Chinese subgroup and overall population in the COMPARZ study. The OS was similar between the pazopanib and sunitinib arms of Chinese subgroup. The efficacy endpoints observed in sunitinib arm of Chinese subgroup were comparable to those reported in previous studies [9–11]. Therefore, the data generated from this Chinese subgroup analysis are relevant to clinical practice.

Overall, the difference of safety between pazopanib and sunitinib arms observed in the Chinese subgroup was similar to overall population in the COMPARZ study. In the Chinese subgroup, hematological toxicities (anemia, leukopenia, lymphopenia, neutropenia, and thrombocytopenia) occurred less frequently in the pazopanib versus sunitinib arm. Hepatobiliary events (increased bilirubin, grade 3 or 4 increased ALT and AST) occurred less frequently in the sunitinib versus pazopanib arm. However, most cases of increased ALT/AST were grade 1/2, and no fatal liver events occurred in the Chinese subgroup. Compared to the results of the overall population in the COMPARZ study, hematological toxicities, hepatobiliary events and fatigue occurred less frequently in Chinese subgroup, while hypertension, HFS, hair color changes occurred more frequently in the Chinese subgroup [8]. The AE profile seen in Chinese subgroup was consistent with the results reported previously for other VEGF TKIs (sunitinib and sorafenib) in clinical practice [11–13]. In the era of immunotherapy, pazopanib and sunitinib are still preferential options in first-line treatment regimens for favorable risk a/mRCC patients, and optimization is more important.

This study has certain limitations. The Chinese subgroup in the COMPARZ study was not randomized. Also, sunitinib treatment regimen in this study was the standard 4/2 schedule (sunitinib 50 mg/day; 4 weeks on treatment, 2 weeks off), while in routine clinical practice, dosing regimens for Chinese patients receiving sunitinib are often adjusted to mitigate toxicity. The small sample size of Chinese patients and limited efficacy analysis as per MSKCC / Heng risk category population are also limitations in this study.

## Conclusions

The efficacy was similar with pazopanib and sunitinib arms in Chinese patients in terms of PFS and OS endpoints, consistent with the overall population in the COMPARZ study. Pazopanib was generally well tolerated in the Chinese population. There were no new safety signals for pazopanib in the Chinese subgroup.

## Data Availability

Novartis is committed to sharing with qualified external researchers, access to patient-level data and supporting clinical documents from eligible studies. These requests are reviewed and approved by an independent review panel on the basis of scientific merit. All data provided are anonymized to respect the privacy of patients who have participated in the trial in line with applicable laws and regulations. This trial data availability is according to the criteria and process described on www.clinicalstudydatarequest.com

## Abbreviations

AE: Adverse event
BIRC: Blinded independent review committee
CI: Confidence interval
HR: Hazard ratio
IRC: Independent review committee
ITT: Intent to treat
mTOR: mammalian target of rapamycin
ORR: Overall response rate
OS: Overall survival
PD: Progressive disease
PFS: Progression-free survival
QD: Once daily
QOL: Quality of life
RCC: Renal cell carcinoma
TKI: Tyrosine kinase inhibitor
VEGFR: Vascular endothelial growth factor receptors
